# Seasonal micro-migration in a farm-island population of striated caracaras (*Phalcoboenus australis*) in the Falkland Islands

**DOI:** 10.1186/s40462-018-0122-8

**Published:** 2018-03-30

**Authors:** Katie J. Harrington, Suzan Pole-Evans, Micky Reeves, Marc Bechard, Melissa Bobowski, David R. Barber, Kalinka Rexer-Huber, Nicolas Lecomte, Keith L. Bildstein

**Affiliations:** 1 Acopian Center for Conservation Learning, Hawk Mountain Sanctuary, 410 Summer Valley Road, Orwigsburg, PA 17961 USA; 2 Moss Landing Marine Laboratories, 8272 Moss Landing Road, Moss Landing, CA 95039 USA; 3 Saunders Island Self Catering, Saunders Island, Falkland Islands; 4 Falklands Conservation, Jubilee Villas, Stanley, Falkland Islands; 5 Department of Biology, Boise State University, Boise, ID 83725 USA; 6 Université de Moncton, Moncton, NB Canada

**Keywords:** Movement ecology, *Phalcoboenus australis*, Island population, Short distance, Seasonal, Space use, GPS, Winter refuge, Marine subsidies, Human subsidies

## Abstract

**Background:**

The extent to which seasonal changes in food availability affect small-scale movements in free-ranging populations of birds of prey is relatively little studied. Here we describe a seasonal “micro-migration” of a farm-island population of striated caracaras (*Phalcoboenus australis*) in the Falkland Islands in response to seasonal changes in the availability of seabird carcasses. We banded more than 450 individuals on Saunders Island, deployed archival and satellite GPS data loggers on 17 individuals, and monitored movements within and between two feeding areas on Saunders Island, a “marine-subsidized” site near seabird colonies and an anthropogenic “human-subsidized” farm site 16 km to the southeast.

**Results:**

During 67 observation days between 2010 and 2015, resightings of 312 banded caracaras were greater at the marine-subsidized site during austral summer than winter, and the total daily resightings varied significantly between spring versus summer, summer versus winter, autumn versus spring, and autumn versus winter. Resightings were higher at the human-subsidized site in austral winter than summer and the total daily resightings varied significantly across all bi-seasonal comparisons. Resightings indicated that at least 12 of 197 birds (6.1%) moved between the human- and marine-subsidized sites at least once during the same winter, 15 of 335 birds (4.5%) did so in spring, none of 164 birds did so in summer, and 16 of 297 birds (5.4%) did so in autumn. Individuals fitted with archival GPS data loggers at the marine-subsidized site in summer maintained highly localized 95% kernel core areas (0.55 ± 0.12 km^2^ [mean ± SD]), whereas those at the human-subsidized site in winter maintained larger 95% kernel core areas (3.8 ± 4.6 km^2^). Two of 6 satellite-tagged individuals that summered at known caracara breeding colonies 80 km WNW of Saunders Island were subsequently resighted in winter at the human-subsidized site.

**Conclusion:**

Our results suggest that seasonal shifts in food resource availability drive seasonal micro-migrations in a farm-island population of striated caracaras, and that farm sites can be critical in providing nutritional resources for caracaras when naturally occurring marine-subsidized resources become less available. Our results have important implications for striated caracara spatial ecology and conservation, as increased winter survival could improve the status of this globally Near-Threatened population.

## Background

Many animals live in seasonally dynamic environments and must adapt accordingly to seasonal changes in their environments. Movement behavior enables such animals to adapt to changes in resource availability in both space and time [1]. The type of movement an animal engages in during such situations depends upon its internal state, its motion and navigation capacities, and both abiotic and biotic external factors [2]. Migration is a response to changes in resource availability over time that involves an animal moving between habitats with seasonally shifting resources [1, 3]. Ball et al. [4] suggests migration strategies fall on a continuum with complete migrant and complete resident behaviors as end points. Within that continuum, strategies vary in terms of distance, frequency, and the proportion of the population undertaking movements [4]. To date, most studied examples of seasonal movements in birds have focused on long-distance migratory movements [1]. Recent research on the European roe deer (*Capreolus capreolus*) [5] supports the notion of a migratory continuum, often over relatively short distances in which individuals commute (sensu [3]) or engage in multiple, brief residencies in seasonal ranges, a movement pattern that includes use of a primary range and a secondary “winter refuge” [5].

Theory predicts that animals should limit time in low-resource areas and move to areas of greater resource abundance [6–8]. Island ecosystems often depend heavily upon allochthonous nutritional inputs from the sea (i.e., “marine subsidies” [9]) that wax and wane seasonally [10, 11], creating times of high and low resource availability. Anthropogenic or “human subsidies” [12] are believed to influence movement at both the individual and population level during times of low resource availability [13, 14]. The degree to which this occurs is not well studied [14]. Here, we explore seasonal short-distance migration of island-restricted species in heterogeneous environments in the Falkland Islands using a diurnal bird of prey, the striated caracara (*Phalcoboenus australis*) as a case study. Specifically, we describe an unusual situation in which striated caracaras move short distances when carcass availability at migratory seabird colonies declines in winter and most of the population moves to seek nutritional resources at a nearby farm site.

Striated caracaras are relatively large, stocky, and inquisitive scavenging and predatory birds of prey (Falconiformes). The species breeds in close proximity to seabird colonies, where individuals feed themselves and their developing young on seabird eggs and nestlings, and on dead and dying adult seabirds [12, 15–17]. Striated caracaras take marine and terrestrial invertebrates [12] and placentas and carrion of terrestrial livestock and marine mammals [15, 18]. Additionally, the species competes for nutritional resources fed upon by turkey vultures (*Cathartes aura*), variable hawks (*Buteo polyosoma*), southern caracaras (*Caracara plancus*), and subantarctic skuas (*Stercorarius antarcticus*) [19].

Striated caracaras are relatively long-lived raptors that obtain adult plumage and begin breeding at approximately five years of age [15]. Before adulthood, individuals often feed in “gangs” on nutritional resources, with up to several dozen young birds competing ravenously for large food carcasses [12, 20].

On the Falklands, traditional sheep farming has largely replaced abundant pinniped populations [15, 17] in winter as a significant nutritional resource for caracaras at a time when most seabirds migrate from the islands. Although some adult caracaras remain near their small breeding territories in winter (MR, pers. obs.), where some switch to feeding largely on invertebrates, others, including many juveniles and sub-adults, leave areas around seabird colonies in winter and spend considerable time in and around farm settlements feeding upon farm scraps [12, 15]. General observations [15] and seasonal body-mass data suggest that caracaras are nutritionally stressed in winter [12]. Unfortunately, despite the globally Near-Threatened status of the species [21], there are few details regarding the specifics of the species’ seasonal movements. Understanding the ecology of these movements has important implications for conservation and management, particularly in light of the fact of historic human-wildlife interactions involving the species at farm settlements [15, 17, 22].

Our objective was to study the behavior of alphanumerically color-marked, mainly juvenile and sub-adult striated caracaras on Saunders Island, a non-breeding “nursery island” for caracaras, to assess the extent of their intra-island movements as individuals switched from feeding on mainly seabirds and tidal invertebrates (i.e., a “marine subsidy” [9]) in summer to feeding on nutritional resources associated with farming (i.e., a “human subsidy” [12]) in winter. To do so, we color banded more than 600 caracaras, placed retrievable GPS data loggers on 11 of them, and tagged 6 individuals with GPS satellite transmitters in 2010–2017.

We predicted (1) that numbers of banded caracaras at the marine-subsidized seabird colonies site would be greater during summer when nutritional resources are plentiful there and lower during winter when marine subsidies are reduced, (2) that numbers of banded caracaras at the human-subsidized farm site would be higher during winter than in summer, (3) that the archival GPS-tracked birds would have small ranges in summer centered around seabird colonies on the island and larger ranges in winter centered around the farm site, and (4) that individuals that left Saunders Island in summer for known caracara breeding sites on islands with seabird colonies might return to Saunders in winter to feed at the human-subsidized farm site.

## Methods

### Study area

**Fig. 1:**
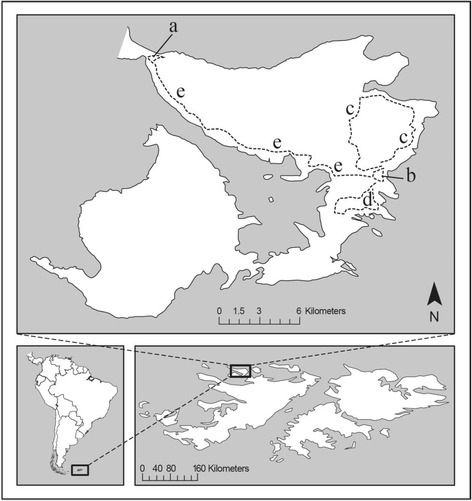
Map of Saunders Island, Falkland Islands, including the two primary study sites (a) the Seabird Colonies Site and (b) the Farm Site, and three all-terrain vehicle (ATV) survey routes: (c) the Egmont Loop, (d) Airstrip Loop, and (e) the Farm Site-to-Seabird Colonies Site Track

Saunders Island (51.37°S 60.09°W) is a 127-km^2^ livestock farm in the northwestern Falkland Island archipelago, 500 km NE of Cape Horn, South America (Fig. 1). Most of the island is hilly and undulating plain covered with dry heath [23]. The farm is operated by a family of four and two co-workers, who maintain approximately 6500 sheep, 200 dairy and beef cattle, 30 goats, 25 horses, 150 chickens, 240 domestic ducks and geese, 2 pigs, and 20 working dogs [12]. The chickens and ducks, free roaming and open-caged, are daily fed grains (approx 28 kg total). The pigs are fed two upland geese (*Chloephaga picta leucoptera*) carcasses (approx. 6 kg) near daily until processed for the family’s personal consumption. The dogs, free roaming and chained, are fed mutton bones nightly (approx. 10 kg total) (KH, pers., comm.).

Our two primary study sites on the island include a 2.5-km^2^ farm settlement (hereafter the “farm site”) where farm operations provide year-round offal, animal feed, compost and other farm scraps; and a 3.5-km^2^ cluster of seabird colonies (hereafter the “seabird colonies site”) at a low-lying 0.35 km-wide isthmus connecting heath-covered uplands 16 km to the northwest of the farm site. Six species of seabirds, including king penguins (*Aptenodytes patagonicus*), gentoo penguins (*Pygoscelis papua*), rockhopper penguins (*Eudyptes chrysocome*), Magellanic penguins (*Spheniscus magellanicus*), imperial shags (*Phalacrocorax atriceps*), and black-browed albatrosses (*Thalassarche melanophrys*), breed at the seabird colonies site in austral summer [12]. The caracaras associate most closely with the gentoo, Magellanic, and rockhopper penguin colonies with estimated population sizes of 2900, 260, and 17,000 individuals, respectively (unpublished observations).

From 2010 through 2015, we caught and banded 459 striated caracaras using monofilament noose traps baited with mutton on Saunders Island. At their time of banding, 59 birds were adults, 46 were sub-adults, 348 were juveniles, and 4 were fledglings, including 113 females, 235 males, and 111 unsexed individuals (Table 1). All captured caracaras were weighed to the nearest 50 g using a hand-held scale, bled for gender analysis, and banded with colored alphanumeric Darvic plastic leg bands. We observed no adverse effects of trapping and handling, with many individuals continuing to feed immediately upon release.

**Table 1:** Summary of banded adult, sub-adult, juvenile, and fledgling striated caracaras 2010–2015 (females, males, unsexed)

	Adult	Sub-adult	Juvenile	Fledgling	ND	Total
2010	0,0,3	0,1,4	1,1,10	0,0,0	2	22
2011	1,7,9	2,4,9	19,28,12	0,0,0	0	91
2012	6,4,4	1,1,1	21,41,1	0,0,0	0	80
2013	2,6,1	1,7,0	23,65,7	0,0,0	0	112
2014	4,6,1	2,6,0	21,49,1	2,1,0	0	93
2015	1,0,4	0,3,4	5,5,38	1,0,0	0	61
All Years	14,23,22	6,22,18	90,189,69	3,1,0	2	459

### Surveys

In December 2011 and then semi-annually through December 2015, we surveyed banded and unbanded birds on five standardized survey routes, including two 2-km transects by foot at the seabird colonies site and the farm site, respectively, and three from all-terrain vehicle (ATV) surveys at 10–20 kph along an additional 33.4 km of the island, including one that circumnavigated Mount Egmont northwest of the farm site (12.1 km), one that traversed an airstrip southeast of the farm site (6.5 km), and one along the principal track between the farm site and the seabird colonies site (14.8 km) (Table 2, Fig. 1). All surveys were conducted between 0800 and 1600 h local time. We recorded the numbers of all banded and un-banded caracaras seen within 200 m of the transects using binoculars and assessed their age by plumage per Strange [15]. Additional observations of banded birds occurred episodically at the farm’s small, open-air pigpen, which in winter can attract over 90 birds while the 1–2 pigs are fed upland geese (SPE pers. obs). We estimate that during the time of our surveys between 60 and 80% of the caracaras on Saunders Island were banded. We did not consider this to be a closed population, as birds tagged with GPS data loggers and GPS satellite transmitters traveled to other islands.

**Table 2:** Total days on which surveys were conducted on Saunders Island 2010–2015

	Spring	Summer	Autumn	Winter
Seabird Colonies Site Transect	24	16	16	13
Farm Site Transect	24	16	16	13
Pigpen Observations	148	31	152	220
Egmont ATV Route	–	13	4	11
Airstrip ATV Route	–	17	9	11
Farm Site-to-Seabird Colonies Site ATV Track	–	14	5	10

### GPS data loggers and satellite tracking

In December 2010 and in July-August 2012 and 2017, we tagged 6 juvenile caracaras at the seabird colonies site and 2 adults, 1 sub-adult, and 2 juveniles at the farm site with battery powered GPS data loggers mounted either backpack-style with a Teflon ribbon harness (cf. [24]) or on their central tail feathers with Tesa Tape. The loggers and harnesses weighed ≤ 40 g, or ≤ 2.7% of a caracara’s body weight, and recorded locations up to once every 10 min. Tagged birds were re-trapped using baited, single-snare traps, their devices removed, and the birds released within 4 min. Data logger tracking periods at the seabird colonies site ranged from 5 to 20 days averaging 9.0 ± 5.2 days (mean ± SD) and from 3 to 9 days averaging 5.8 ± 2.5 days at the farm site.

In 2013, we successfully deployed six 30 g solar-powered GPS-PTTs (Microwave Telemetry, Inc., Columbia, MD, USA), mounted backpack-style with Teflon ribbon harnesses (cf. [24]), on 1 adult, 4 sub-adult, and 1 juvenile caracara. Three were deployed at the Saunders Island Settlement and three were deployed at the Carcass Island Settlement 34 km to the WNW of the Saunders Island farm site banding location. We received transmissions hourly from 0500 to 2200 local time.

All manipulations were conducted in accordance with the Falkland Island Government Conservation of Wildlife and Nature Ordinance under Research License No: R22/2015.

### Movement analysis

We restricted our banding resighting analyses to individuals resighted in seasons subsequent to the one in which they were banded to allow individuals time to move within the island. All resightings of banded caracaras were categorized by year (2012–2015) and austral season, (e.g. spring [September-November], summer [December-February], autumn [March-May], and winter [June-August]).

To avoid biasing our results because of greater resighting effort at the farm site, we restricted our analysis to the 67 days when resightings were undertaken at both the seabird colonies site and the farm site. We used R v. 1.0.136 for statistical analyses, using Kruskal-Wallis tests to evaluate seasonal differences in abundance, as parametric assumptions were not met, and Dunn’s test of multiple comparisons for post-hoc tests.

We separately analyzed 8700 resightings of banded birds obtained during 515 observation days at pig feedings at the farm site to assess seasonal patterns that might not have been reflected in the 67 days of equal two-site effort.

We calculated Minimum Convex Polygons (MCP) and both 95 and 50% kernel core areas using all recorded locations of the 11 GPS-tagged birds (Kernel Density Estimator, Spatial Analyst Toolbox, ArcMap, ArcGIS 10.4.1).

## Results

**Fig. 2:**
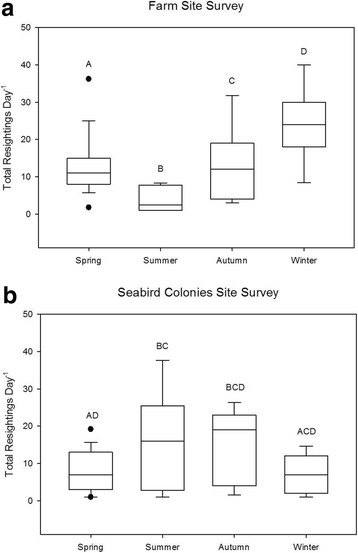
Differences in total resightings per day by season at (**a**) the Farm Site and (**b**) the Seabird Colonies Site, Aug 2012-Mar 2015. The lower boundary of the box indicates the 25th percentile, the line within the box marks the median, and the upper boundary of the box indicates the 75th percentile. Whiskers indicate the 95th and 5th percentiles. Dots represent outliers. Different letters indicate statistical differences among seasons

During 67 observations days across 16 seasons at both the farm site and the seabird colonies site, we resighted 281 different color banded individuals, including 105 males, 151 females, and 25 birds of unknown sex. See Table 2 for seasonal sample sizes per transect. Resightings indicated that 11 of 196 birds (5.6%) moved between the farm site and seabird colonies site at least once during the same winter, that 14 of 334 birds (4.2%) resighted in spring did so, that none of 164 birds seen in summer did so, and that 16 of 297 birds (5.4%) resighted in autumn did so. Overall, there was a statistically significant difference in total resightings per day among seasons at both the seabird colonies site (χ^2^ = 9.60, df = 3, *p* < 0.05) and the farm site (χ^2^ = 32.33, df = 3, *p* < 0.01). Caracara resightings at the seabird colonies site were highest during austral summer (15.9 resightings per day) and lowest in austral winter (7.3 resightings per day) (Fig. 2a); in contrast, farm site resightings were highest during austral winter (24.4 resightings per day) and lowest during austral summer (3.9 resightings per day) (Fig. 2b). A post-hoc Dunn’s test showed total resightings varied significantly across seasons at the farm site (*p* < 0.01, Table 3), and that total resightings at the seabird colonies site varied significantly between spring versus summer, summer versus winter, autumn versus spring, and autumn versus winter (*p* < 0.05, Table 3).

**Table 3:** *P*-values for Dunn’s test of multiple comparisons using rank sums^a, b^

Location	Autumn vs. spring	Autumn vs. summer	Spring vs. summer	Autumn vs. winter	Spring vs. winter	Summer vs. winter
Seabird Colonies Site Transect	*	0.8	*	*	0.9	*
Farm Site Transect	**	**	**	**	**	**
Pigpen Observations	**	**	**	**	**	**
Egmont ATV Route	ID^b^	*	ID	0.7	ID	**
Airstrip ATV Route	ID	*	ID	0.2	ID	**
Farm Site-to-Seabird Colonies ATV Track	ID	**	ID	0.8	ID	**

^a^Single asterisks indicate *P*-values of ≤0.05. Double asterisks indicate *P*-values of < 0.01

^b^ID indicates insufficient data for analysis. See Table 2 for sample sizes

**Fig. 3:**
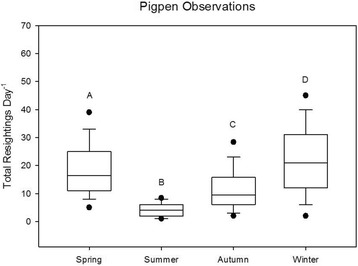
Differences in total resightings per day by season of striated caracara during pigpen observations, Jul 2011-May 2015. The lower boundary of the box indicates the 25th percentile, the line within the box marks the median, and the upper boundary of the box indicates the 75th percentile. Whiskers indicate the 95th and 5th percentiles. Dots represent outliers. Different letters indicate statistical differences among seasons

We resighted 305 individuals, including 123 males, 146 females, and 36 of unknown sex during 515 observation days at the pigpen feeding site. There was a statistically significant difference in daily resighting rates among seasons (χ^2^ = 127.96, df = 3, *p* < 0.01) (Fig. 3), with caracara numbers being highest (22.0 resightings per day) at the pigpen during winter and lowest during summer (3.9 resightings per day). A post-hoc Dunn’s test showed total resightings varied significantly across seasons (*p* < 0.01, Table 3).

**Fig. 4:**
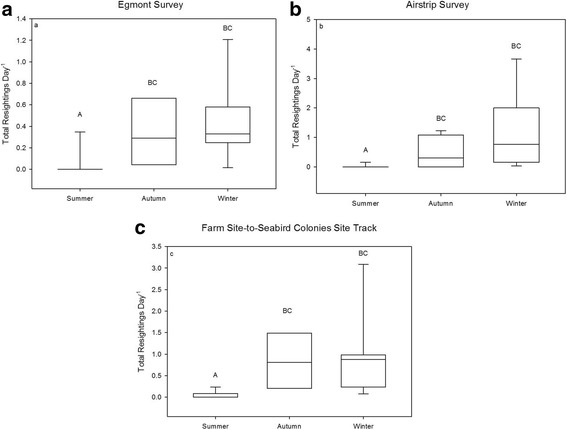
Differences in total resightings per day by season of striated caracaras from our three ATV surveys: (**a**) the Egmont loop, (**b**) the Airstrip loop, and (**c**) the Farm Site to Seabird Colonies Site track (*n* = 26, *n* = 37, *n* = 29 respectively). The lower boundary of the box indicates the 25th percentile, the line within the box marks the median, and the upper boundary of the box indicates the 75th percentile. Whiskers indicate the 95th and 5th percentiles. Dots represent outliers. Different letters indicate statistical differences among seasons

There was a statistically significant difference in resightings among seasons on all ATV survey routes (χ^2^ = 12.075, df = 2, *p* < 0.01; χ^2^ = 18.611, df = 2, *p* < 0.01; χ^2^ = 15.682, df = 2, *p* < 0.01; Egmont Route, Airstrip Route, and Farm Site-to-Seabird Colonies Site Track, respectively, Fig. 3). Our highest resightings on the Egmont and Airstrip ATV surveys occurred during winter (0.4 birds km^− 1^ and 1.2 birds km^− 1^, respectively) (Fig. 4a and b), with the majority of resightings happening at or within 1.5 km of the start and end points of the routes that were adjacent the farm site. Our highest caracara counts on the Farm Site-to-Seabird Colonies Site Track occurred during winter (0.9 birds km^− 1^) (Fig. 4c), with the majority of resightings clustered within 1.5 km of the farm site. A post-hoc Dunn’s test showed total resightings varied significantly from autumn to summer and summer to winter for all ATV surveys (*p* < 0.05, Table 4).

**Table 4:** Minimum Convex Polygons (MCP) and kernel core areas (km^2^) for GPS-tagged individuals

Location	100% MCP	95%	50%
Seabird colonies site (*n* = 6)	1.73 ± 0.47	0.55 ± 0.12	0.05 ± 0.02
Farm site (*n* = 5)	28.03 ± 19.29	3.85 ± 4.56	0.16 ± 0.30

Six birds fitted with retrievable GPS data loggers at the seabird colonies site maintained relatively small 100% MCP areas of use (1.73 ± 0.47 km^2^ [mean ± SD]) at the site during 5–20 days. Five birds fitted with retrievable GPS data loggers at the farm site maintained larger 100% MCP areas of use (28.03 ± 19.29 km^2^) over 3–9 days at the site (Table 4). Removing 5% of all outlier positions using the quadratic kernel function [25] resulted in 95% kernel core areas of 0.55 ± 0.12 km^2^ at the seabird colonies site and 3.85 ± 4.56 km^2^ at the farm site.

Three of the six satellite-tagged individuals summered at known breeding colonies on Grand Jason and Steeple Jason Islands 80 km WNW of Saunders Island. Of those, two were subsequently resighted in winter at the human-subsidized farm site, whereas the third individual’s unit failed on Steeple Jason Island. Of the remaining three individuals, one remained on Saunders Island year round with periodic visits of < 7 days during autumn, winter, and spring, to Carcass Island and West Falkland Island, whereas two individuals removed their units after 2 and 4 months.

## Discussion

Overall, the unusual micro-migration of this farm-island population demonstrates the extent to which flexibility exists within the movement ecology of this species. Our study describes short-distance, seasonal to-and-fro movements of a farm-island population of striated caracaras between marine-subsidized ranging areas near seabird colonies of king, gentoo, rockhopper, and Magellanic penguins, imperial shags and black-browed albatrosses, and human-subsidized ranging areas at a farm site 16 km east-southeast of the seabird colonies site on 127-km^2^ Saunders Island in the Falklands Islands. Anecdotal reports have long suggested that striated caracaras are attracted to and use farm sites on the Falklands as winter refuges (sensu [5]) [15, 17]. We build on preliminary work [12] to describe in detail the timing and extent of use of these seasonally significant feeding sites, as well as the geographic areas over which individual caracaras are attracted to them.

Striated caracaras are aggressive and opportunistic scavenging birds of prey that breed singly and in colonies near and on the perimeters of large colonies of nesting seabirds where they feed upon seabird eggs, nestlings, and vulnerable adult seabirds, as well as upon the nearby carcasses and faeces of marine mammals and livestock carcasses [15]. Our summer fieldwork indicates that caracaras also routinely feed upon beach and marine invertebrates, including kelp maggots (Diptera) and limpets (*Patinigera* sp.), and on at least one occasion, caracaras were observed eating an octopus (*Enteroctopus megalocyathus*) (KJH and KLB, pers. obs.).

Breeding success is high in striated caracaras in the Falkland Islands [22], and food stress, characteristic of their winter condition, particularly among first-year birds, is likely to be a limiting factor of population growth [12, 15, 26]. Most of the seabirds that form the bulk of the caracaras’ summertime diet are intermediate to long-distance migrants (e.g., Magellanic and rockhopper penguins) [27, 28] that leave the Falklands in autumn and return in spring [22]. Importantly, caracaras are not considered migratory on the Falklands and remain on the archipelago year-round [15, 22].

Historically, overwintering caracaras are believed to have depended heavily upon the carcasses and feces of pinniped populations that bred on the islands prior to the latter’s decimation by sealers during an extended “seal rush” during late 1700s and mid 1800s [15, 29]. Since the demise of pinniped populations in the nineteenth century, overwintering populations of striated caracaras have increasingly exploited nutritional resources found at and near human settlements where human trash, including the remains of slaughtered livestock, provide offal and farm scraps for the birds [15]. In addition, improved, high-quality pasturage surrounding farm sites concentrates both livestock and upland geese, which when preyed upon by variable hawks [12] provide carcasses for the caracaras, the former via increased overwinter mortality, and the latter when geese are preyed upon by variable hawks [12], that are voraciously competed for by “gangs” of caracaras [20].

Our principal study site, Saunders Island, is not a breeding site for caracaras, but rather is a non-breeding nursery island for a population of more than 100 caracaras, most of which are juveniles and sub-adults. A lesser number of adult caracaras on the island include year-round, non-breeding residents (KLB, pers. obs.) and adult breeders that return from breeding sites on other islands to overwinter at the farm site on Saunders. Our observations indicate that the seasonal to-and-fro movements of caracaras between seabird colonies on the island and the island’s farm site are best characterized as instances of “partial migration” (sensu [5]) in which most caracaras move from a principal home range near seabird colonies in summer, early-autumn, and late-spring, to secondary feeding sites in and around a winter refuge farm site. While we have not quantified seasonal differences in foraging success, body masses of male and female striated caracaras on Saunders Island collected in 2011 and 2012 indicate that both male caracaras (which are smaller than females) and female caracaras weigh 14–15% less in winter at the farm site than in summer at seabird colonies [12]. This suggests that the increased use of the farm site in winter is not due to asymmetric seasonal shifts in food availability at the two sites, but rather is due to the birds making the best of a bad situation by moving to lower food availability at the farm site only when food availability at the seabird colonies site drops below that available at the winter refuge. In winter, the seabird population at the colonies site on Saunders Island declines by an estimated 85%, while the farm site food availability is maintained year-round. The two foraging areas, which are 16 km apart, do not differ altitudinally (both are less than 10 m in altitude), nor climatically (both are less than 500 m from the island’s coastline). The two sites, however, do differ in that one offers a summertime “rich patch” (sensu [30]) or nutritional marine subsidy in the form of migratory seabirds and their eggs and young, and the other offers a nutritionally less adequate, but year-round “human subsidy” in the form of farm scraps and carcasses associated with farming [12].

To our knowledge this type of non-altitudinal, short-distance, “micro-migration,” has not been reported in other birds, however, a somewhat similar pattern has been described in at least one mammal. In Europe, GPS-tracked roe deer (*Capreolus capreolus*) exhibit partial to-and-fro migration in which some but not all individuals migrate to-and fro short-distances between principal home ranges and episodic winter refuges [5]. That caracaras do so on Saunders Island likely is enhanced by the fact that within seasons, inter-site journeys can be undertaken in less than a few hours, with two-way commutes between the two sites occurring occasionally, even on successive days.

### Conservation implications

Understanding the extent to which a species may benefit nutritionally from human subsidies during times of nutritional stress is critical to understanding the impact of human actions on that species’ conservation status. Understanding risks posed to a species when using such human-dominated landscapes is equally crucial. Strange 1996 [15], Woods [17], and the observations presented here suggest that at least some caracaras in the Falklands exploit human subsidies at farm sites in winter (cf. [12]). That Saunders Island is a non-breeding nursery island suggests juvenile survival could be increased by these human subsidies and that farm sites in the Falklands may provide resilience to the Falklands population of striated caracaras.

## Conclusions

Most avian migrations, including those of birds of prey, entail multi-day, if not multi- week or multi-month journeys, many of which involve long-distance linearized movements, requiring pre-migratory fat loading, refueling en route, atmospheric-assisted soaring flight, or all three methods of assistances [31, 32]. The seasonal to-and-fro movements of caracaras described above do not fit this model, but could instead be called “micro-migrations.” Migration has been characterized as a behavioral tool in which “migrants leave habitats where resources are deteriorating or their availability is otherwise reduced to colonize or take refuge in habitats were resources are available at least for maintenance” [1]. In this regard, the seasonal movement of the bulk of the population of caracaras on Saunders Island between summering and wintering areas reflects an extremely flexible and sometimes reversible form of migratory behavior, micro-migration, the fitness consequences of which have yet to be studied in detail. A careful examination of long-term movements of individually marked birds coupled with individual variations in their lifetime breeding successes and long-term survival is needed. Such a study is now underway on Saunders Island.

## Abbreviations

ATV: All terrain vehicle
GPS: Global positioning system
MCP: Minimum Convex Polygon
PTT: Platform transmitter terminal
